# Extraskeletal myxoid chondrosarcoma: A report of two cases

**DOI:** 10.3892/ol.2014.1884

**Published:** 2014-02-14

**Authors:** GUO-WEI ZHANG, AI-JIE WANG, GUANG-HUI ZHANG, SHAN-NA ZHAO, JIAN-LI QU

**Affiliations:** CT/MR Room, Yantaishan Hospital, Yantai, Shandong 264001, P.R. China

**Keywords:** extraskeletal myxoid chondrosarcoma, buttock, knee

## Abstract

Extraskeletal myxoid chondrosarcoma (EMC) is a relatively rare but well-defined neoplasm. This report describes two patients, one with EMC of the buttock and one with EMC of the knee. The two cases presented with large lobed masses and long T1 and T2 signaling identified by magnetic resonance imaging (MRI). An enhanced MRI scan demonstrated enhancement of the tumors. The tumors were composed of strands or cords of oval and spindle cells embedded in abundant myxoid stroma. Pathology results confirmed EMC.

## Introduction

Extraskeletal chondrosarcomas were first described by Stout and Verner in 1953 (1); however, it was not until 1972 that extraskeletal myxoid chondrosarcoma (EMC) was histopathologically defined as its own entity (2). EMC is provisionally classified as a tumor of uncertain differentiation in the revised version of the World Health Organization classification of tumors of soft tissue and bone in 2002 (3). EMC is a relatively rare but well-characterized tumor that accounts for <2% of all soft tissue sarcomas (4). Approximately 80% of these tumors occur in the extremities, with 20% located in the trunk. The lower extremity is the most common location of EMC (4). The male to female ratio of EMC is 2:1, with a peak occurrence in the fifth and sixth decades (4).

This report presents two patients, one with EMC of the buttock and the other with EMC of the knee. Both patients provided written informed consent.

## Case reports

### Case 1

**Figure 1:**
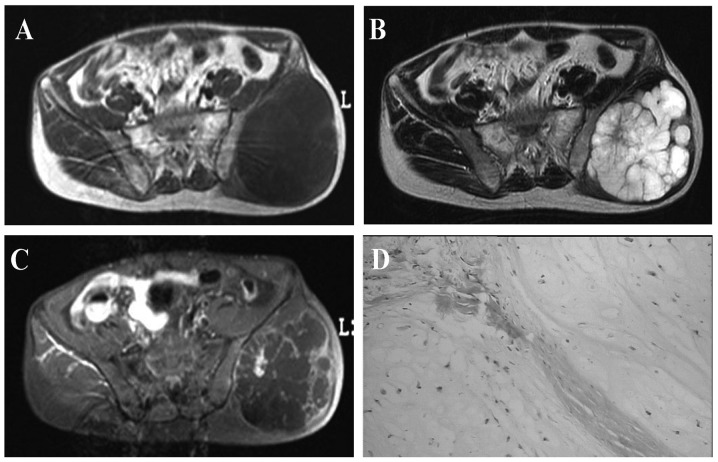
MRI findings and histological features of the patient in case 1. (A) T1-weighted MRI shows a uniform low signal in T1WI. (B) T2-weighted MRI shows a heterogeneous high signal in T2WI. (C) T1-weighted enhanced MRI shows strong enhancement of the tumor. (D) The tumor consists of strands or cords of oval cells and abundant myxoid stroma (hematoxylin and eosin; magnification, ×200). MRI, magnetic resonance imaging; WI, weighted image.

A 47-year-old man presented with a five-year history of a painless lump in his left buttock. Six months prior to presentation, the patient had noted that the mass had gradually enlarged and become hard. Magnetic resonance imaging (MRI) revealed an 8×6×6-cm sized lobular mass in the left buttock that had long T1 and T2 signal intensities. The MRI scan also revealed a uniform low signal in the T1 weighted image (WI) (Fig. 1A) and a high signal in the T2WI, with fat suppression and uneven signaling inside the mass and radiated arrangement of low signaling separation in the middle of the mass (Fig. 1B). An enhanced MRI scan revealed an obvious and uneven enhanced mass with radiated point bar enhancement in the middle of the mass in T1WI (Fig. 1C). In addition, the adjacent bone showed normal signaling with mild edema of the surrounding soft tissue. During surgery, a mass outside the left iliac bone plate was identified. The mass comprised jelly-like tissue inside and adhered to the surrounding sciatic nerve. The physician excised the mass, which was located in the subcutaneous tissue and consisted of chondroid tissue with lobes and nodular arrangement. A tissue specimen was then sent to the pathologist for analysis. The tumor was found to be composed of strands or cords of oval and spindle cells embedded in abundant myxoid stroma (Fig. 1D). Pathological analysis of the specimen concluded that the tumor was myxoid chondrosarcoma.

### Case 2

**Figure 2:**
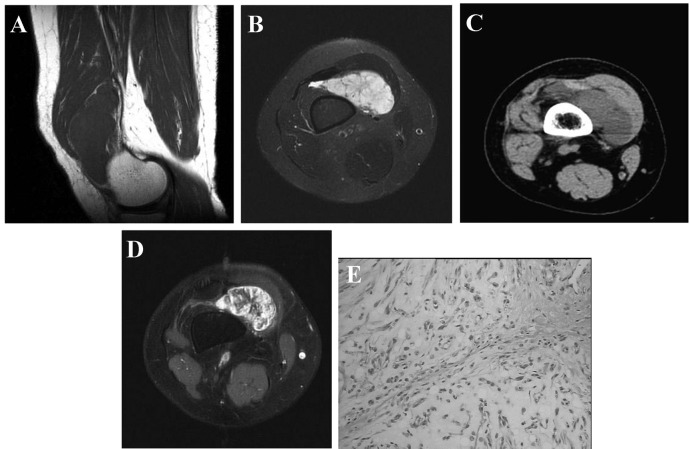
Imaging findings and histological features of the patient in case 2. (A) CT reveals an irregular-shaped soft tissue mass measuring 8.0×6.6×3.3 cm in size in the right knee bursa, and the mass shows a clear boundary and uniform density. The CT value was ~23 HU. (B) T1-weighted MRI shows a uniform low signal in T1WI. (C) T2-weighted MRI shows a heterogeneous high signal in T2WI. (D) T1-weighted enhanced MRI shows strong enhancement of the tumor. (E) The tumor consists of strands or cords of oval cells and abundant myxoid stroma (hematoxylin and eosin; magnification, ×200). CT, computed tomography; MRI, magnetic resonance imaging; WI, weighted image.

A 45-year-old woman presented with a painless lump in her right knee for one week. Physical examination demonstrated that the right lower extremities and knee were slightly swollen with a palpable mass, but the patient had no difficulty in mobilization. Computed tomography (CT) revealed an irregular-shaped soft tissue mass measuring 8.0×6.6×3.3 cm in size located in the right knee bursa, and the mass showed clear boundary and uniform density (Fig. 2A). The adjacent bone of the mass did not show obvious absorption and destruction. MRI confirmed the presence of an irregular-shaped soft tissue mass in right knee bursa with long T1 and T2 signaling, as well as a uniformed low signal in T1WI (Fig. 2B) and a high signal with fat suppression in T2WI (Fig. 2C). An enhanced MRI scan revealed an obvious uneven enhanced mass with radiated arrangement separation enhanced like spokes in T1WI (Fig. 2D). During surgery, a mass was located in the deep surface of the rectus femoris and vastus lateralis. The mass had the appearance of pale yellow soft tissue and was brittle with a large quantity of mucus. Histological examination identified that the tumor was composed of clustered and trabecular-shaped cells in an abundant myxoid matrix. The tumor cells had relatively uniform oval nuclei with dense, evenly dispersed chromatin and a moderate amount of eosinophilic cytoplasm that was often finely vacuolated (Fig. 2E). Immunohistochemical stains showed that the tumor cells were negative for smooth-muscle actin (SMA), myogenin and CKpan, and positive for S-100 and vimentin. Based on these findings, the patient was diagnosed with EMC.

## Discussion

EMC is a relatively rare neoplasm with no specific findings in the clinic. Patients commonly present with non-specific symptoms, including tenderness and the detection of a palpable mass (3). The most common manifestation of EMC is an enlarging soft tissue mass; some lesions are accompanied by pain and tenderness, or may restrict the range of motion. Long-term follow-up studies have shown that EMC is a slowly growing tumor with a risk of local recurrence or distant metastasis and disease-associated mortality (3). The lesions exhibit low density on CT, low signal intensity on T1-weighted MRI scans and a high signal intensity on T2-weighted MRI scans (6). Microscopically, the tumors are characterized by a proliferation of ovoid and bipolar cells that are enmeshed in a prominent myxoid matrix rich in chondroitin and keratin sulfate (7,8). Immunohistochemically, the neoplastic cells commonly stain with antibodies to vimentin and S-100 protein. Certain studies have shown that they may also be positive for Leu-7 and epithelial membrane antigen. Uniformly, they are negative for keratin, SMA and desmin (9,10).

This study describes two patients, one with EMC of the buttock and one with EMC of the knee. EMC of the buttock has rarely been reported; since its first description in 1972 (2), only a small number of cases have been discussed. The two cases presented in this report demonstrated large lobed masses and long T1 and T2 signals on MRI. An enhanced MRI scan showed enhancement of the tumors. The tumors were found to be composed of strands or cords of oval and spindle cells embedded in abundant myxoid stroma.

The differential diagnosis of EMC is broad and includes mucus liposarcomas and soft tissue myxomas. Mucus liposarcomas often present as a large bump situated in the muscles, with a clear boundary, multilocular high signaling in T2WI and without radial low signal separation. Soft tissue myxomas belong to the embryonic mesenchymal benign tumors. The appearance in MRI of an intramuscular lesion with low T1 signal and high signal intensity on fluid-sensitive sequences demonstrating a peripheral rim of fat and edema is highly suggestive of a soft tissue myxoma (11). The tumors present with a clear boundary on CT scans, with uniform density and without calcification. MRI often shows long T1 and T2 signaling, and no obvious enhancement. In conclusion, when soft tissue masses exhibit significantly long T1 and T2 signal intensities, lesions appear in radial short T2 signal separation and an enhanced MRI scan reveals enhancement of tumors, EMC should be considered as a possible diagnosis.
